# Editorial: Gut physiology—microbes and inflammatory diseases

**DOI:** 10.3389/fphys.2023.1254228

**Published:** 2023-07-21

**Authors:** Shubha Priyamvada, Suhail Akhtar

**Affiliations:** ^1^ Department of Medicine, University of Illinois at Chicago, Chicago, IL, United States; ^2^ Department of Biochemistry, A.T. Still University, Kirksville, MO, United States

**Keywords:** microbiome, IBD, gut physiology, host-microbe interaction, intestine, dysbiosis, inflammation

Gut microbiome though physically contained within the gut lumen is capable of exerting far reaching effects (Priyadarshini et al., 2018) regulating overall host physiology-metabolism, immunity and development (Aron-Wisnewsky et al., 2021). This contribution of the gut microbiome to host health is either through the production of a myriad of metabolites or its interaction with the gut epithelium and associated lymphoid tissue (Kayama et al., 2020). At the same time, host-endogenous and host-exogenous factors impact the composition and the function of the gut microbiome (Kayama et al., 2020). This plasticity of the gut microbiome has allowed its evaluation as a causative as well as therapeutic target in several pathophysiological states. Despite the significant advances made in the techniques studying the role of gut microbiome in regulating different aspects of host health, its causal role in a disease’s progression or remission is still not completely deciphered and is being researched rigorously. Our Research Topic brings forth some latest articles in the field.

Among the gut microbial metabolites, the short chain fatty acids are the most commonly studied, and notable for their beneficial effects (Krautkramer et al., 2021). One short chain fatty acid, butyrate, serves as a major energy source for the colonocytes, educates both innate lymphoid cells and adaptive immune cells, and facilitates intestinal epithelial cell proliferation by regulating the cell cycle (Kayama et al., 2020). These effects are not limited to the gut. Butyrate exerts systemic effects too like increasing insulin sensitivity, and lowering lipid levels, combating lung inflammation, preventing surgery induced arterial injury and autoimmune diabetic islet damage (Sun et al., 2015; Vieira et al., 2019; Nooromid et al., 2020; Krautkramer et al., 2021). Describing similar benefits of butyrate in an understudied area of acute pancreatitis Xiong et al. have shown that butyrate intervention preserved gut barrier integrity by maintaining tight junction protein expression and reduced pancreatic immune cell infiltration. Earlier research has also highlighted that butyrate mediated histone deacetylase inhibition maintains intestinal barrier by regulating the expression of cytokines and mucins (Kayama et al., 2020). While histone deacetylase (HDAC) inhibition was not explored by Xiong et al. a similar mechanism is expected (Bordin et al., 2004). In fact, by the virtue of its most potent HDAC inhibitor properties, butyrate occupies a unique stand among other short chain fatty acids. Xiong et al. also describe butyrate induced changes in the gut microbiota promoting anti-inflammatory, short chain fatty acid producing genera. Thus, butyrate intervention reversed the dysbiosis and overall reduced the disease pathology.

Alterations in the functionality and composition of the gut microbiome are implicated in several disease pathologies including most debilitating intestinal diseases, inflammatory bowel disease and colorectal cancer (Byndloss et al., 2017; Wong and Yu, 2019; Krautkramer et al., 2021; Priyadarshini et al., 2022). Role of gut microbiome in colorectal cancer is increasingly recognized from contribution of specific microbial oncogenic metabolites/factors to collective microbial community alterations promoting growth of procarcinogenic and/or opportunistic passenger bacteria (Wong and Yu, 2019). Colorectal cancer patients also have signature metabolomic profiles (Wong and Yu, 2019). Enumerating similar gut microbial changes in a distinct cohort of colorectal cancer subjects Du et al. showed inverse association between enriched gut bacterial strains and fecal monosaccharides in the diseased subjects. Restoration of eubiosis (a balanced health promoting microbial community) could thus be a therapeutic strategy against diseases associated with dysbiosis. However, research is needed to investigate ways to modulate gut microbiota. In a similar quest in their original research article, Liang et al. explored gut microbiome restorative potential of polysaccharides extracted from a traditional herbal medicine *Dendrobium candidum*. Far reaching effects of the gut microbiome are re-affirmed in their model of atopic dermatitis where restoration of normal gut microbiome promoted not only intestinal homeostasis but also mitigated the symptoms of the disease. Biliary disease is another example of gut microbiome and remote organ crosstalk (Hu et al., 2022; Dan et al., 2023). High incidence of biliary diseases imposes significant health and financial burden. In their research article, Zhang et al. offer comparative evaluation of endoscopic retrograde cholangiopancreatography and laparoscopic transcystic common bile duct exploration approaches in the treatment of bile duct stones. Articles covered under this Research Topic indicate the role of the communication between endogenous gut microbial members and host tissues and suggest the potential of microbiota modulation strategies as therapeutic tools. We hope you share our excitement in reading these articles.
